# Prevalence and risk factors of prehypertension/hypertension among freshman students from the Vietnam National University: a cross-sectional study

**DOI:** 10.1186/s12889-023-16118-4

**Published:** 2023-06-16

**Authors:** Hong-Khoi Vo, Dung Viet Nguyen, Thom Thi Vu, Hieu Ba Tran, Hoai Thi Thu Nguyen

**Affiliations:** 1 Neurology Center, Bach Mai Hospital, Hanoi, Vietnam; 2 Department of Neurology, VNU-University of Medicine and Pharmacy, Hanoi, Vietnam; 3 Department of Internal Medicine, VNU-University of Medicine and Pharmacy, Hanoi, Vietnam; 4 Vietnam National Heart Institute, Bach Mai Hospital, Hanoi, Vietnam; 5 Department of Basic Medical Sciences, VNU-University of Medicine and Pharmacy, Hanoi, Vietnam

**Keywords:** Hypertension, Prehypertension, Risk factors, Students, Young adults, VNU, Vietnam

## Abstract

**Background:**

Prehypertension (PHT) and hypertension (HTN) in young adults are essential risk factors for other cardiovascular diseases (CVD) in later years of life. However, there is a lack of knowledge about the burden and risk factors of PHT/HTN for Vietnamese youth. The aim of this study was to investigate the prevalence of PHT/HTN and risk factors among university students in Hanoi, Vietnam.

**Methods:**

This study was designed as a cross-sectional investigation with 840 students (394 males and 446 females) randomly sampled from freshmen of Vietnam National University, Hanoi (VNU). Socio-demographic, anthropometric, and lifestyle data were collected using questionnaire forms and physical measurements. HTN was defined as blood pressure (BP) ≥ 140/90 mmHg and/or current treatment with antihypertensive medications. PHT was defined as a systolic BP from 120 to 139 mmHg and/or a diastolic BP from 80 to 89 mmHg. Body mass index (BMI) was classified according to the WHO diagnostic criteria for Asian adults: normal weight (BMI 18.5–22.9 kg/m^2^), underweight (BMI < 18.5 kg/m^2^), overweight (BMI 23–24.9 kg/m^2^), and obese (BMI ≥ 25 kg/m^2^). Bivariable and multivariable log-binomial regression analyses were conducted to explore the association of PHT/HTN with different risk factors.

**Results:**

The overall prevalence of prehypertension and hypertension was 33.5% [95% CI: 30.3–36.8%] (54.1% in men and 15.3% in women) and 1.4% [95% CI: 0.7–2.5%] (2.5% in men and 0.5% in women), respectively. Regarding CVD major risk factors, 119 (14.2%) were identified as overweight/obese, 461 (54.9%) were physical inactivity, 29.4% of men and 8.1% of women reported consuming alcohol. The multivariable analysis indicated the male sex (adjusted prevalence ratio [aPR] = 3.07; 95% CI: 2.32–4.06), alcohol consumption (aPR = 1.28; 95% CI: 1.03–1.59) and obesity (aPR = 1.35; 95% CI: 1.08–1.68) as the independent risk factors for PHT/HTN.

**Conclusions:**

The results revealed the high burden of prehypertension and hypertension among university freshmen in VNU. Male sex, alcohol consumption, and obesity were identified as important risk factors for PHT/HTN. Our study suggests an early screening program for PHT/HTN and campaigns to promote a healthy lifestyle for young adults in Vietnam.

**Supplementary Information:**

The online version contains supplementary material available at 10.1186/s12889-023-16118-4.

## Background

Cardiovascular diseases (CVDs) are currently the leading cause of morbidity and mortality worldwide and disease burden globally, accounting for approximately 18 million deaths per year [1, 2]. Hypertension affects one-third of the worldwide population and is the leading modifiable risk factor for several CVDs, such as cerebrovascular disease, heart failure, ischemic heart diseases, and peripheral arterial diseases [3, 4]. Although hypertension (HTN) is conventionally more common among elderly individuals, recent epidemiological studies have revealed that hypertension and prehypertension (PHT) may initiate during adolescence and persist into adulthood [5]. Furthermore, the earlier the onset of PHT and HTN, the higher the risk for other CVDs and mortality [6, 7].

In Vietnam, the majority of epidemiological investigations have concentrated on the prevalence of PHT/HTN and their associated risk factors in the general population. However, there is a lack of data on the prevalence of PHT/HTN and related factors among young Vietnamese people, especially those studying at universities.

The aim of this study was to evaluate the prevalence of PHT/HTN and investigate their potential risk factors among college freshmen at Vietnam National University, which would contribute to the evidence on preventive cardiology. Vietnam National University is a multidisciplinary university consisting of multiple member colleges/faculties, along with a large number of students admitted from all regions of the country. Therefore, the ability to generalize the research findings obtained from this study will be at an acceptable level.

## Methods

### Study design and setting

The study was designed as a cross-sectional investigation conducted at Vietnam National University, Hanoi (VNU), from December 2016 to January 2017. VNU is the leading center for comprehensive education, instruction, and research in Vietnam, with 21 member colleges and faculties, which include more than 48,000 students (with approximately 48% male and 52% female). Every year, VNU graduates over 6,000 bachelors, 2,000 masters, and 200 doctors from a multitude of undergraduate, master, and doctoral programs in various fields such as natural sciences, law, medicine, engineering, business, computing, and social sciences.

### Study participants and sampling

All college freshmen of the VNU were the source population. Students who were at least 18 years of age and did not have any severe chronic illnesses were considered to meet the inclusion criteria. Severe chronic illnesses were defined as chronic diseases that potentially impact blood pressure (e.g., kidney disease, diabetes, hormone problems, lupus, scleroderma) or health conditions require ongoing medical treatment which can affect blood pressure (e.g., steroids, NSAIDs, antidepressants, contraceptive pill), or both. The study excluded pregnant or breastfeeding women and participants with cancer or cardiovascular diseases, serving as the exclusion criteria. All students who met the criteria for eligibility and consented to the study were included in our study.

The sample size n for the prevalence objective was calculated using the following formula: n = N*X / (X + N – 1), where X = Z_α/2_^2^*p*(1-p) / MOE^2^ (with α = confidence level; p = estimated proportion of prehypertension/hypertension among VNU freshmen; MOE = the margin of error; N = the total freshmen) [8]. Choose a confidence interval (CI) of 95%, an estimated MOE of 3%, p = 29.3%, N = 6540; the required sample size was 779 students [9]. To account for potential non-response, we added 10% to the required sample, resulting in a final estimated sample size of 857.

The participants were selected using simple random sampling. First, a sampling frame was created by obtaining a list of all first-year students enrolled at Vietnam National University. A software (STATA) was then used to generate a list of 857 random students from the sampling frame. The corresponding students were invited to participate in the study via mobile phone. During the phone contact process, if any student on the list could not be reached after three calls over three consecutive days, did not meet the selection/exclusion criteria (based on information gathered via phone), or declined to participate in the physical examination and direct interview, we repeated the random sampling process to select another student as a replacement. In the end, a total of 857 students agreed to participate in the physical examination and direct interview when contacted via phone. Of those, 840 students met all the criteria for participation in the study, agreed to sign the informed consent form, and were included in the final analysis.

### Data collection

All participants were asked to complete a standard-structured questionnaire and underwent a mandatory physical examination by trained physicians. The questionnaire included two primary sections to gather information on socio-demographic characteristics and related factors for PHT/HTN, including cigarette and alcohol use, family history of HTN, physical activity, and consumption of fruit, vegetable, and salt.

Subjects were instructed to avoid drinking, cigarette smoking, exercising, tea, and coffee for at least 30 min prior to the blood pressure measurement. Throughout the examination, blood pressure (BP) was measured three times consecutively per participant with an interval of no less than 2 min by an attending doctor using an oscillometric electronic sphygmomanometer (HEM-6221; Omron, Kyoto, Japan), with the participants in a seated position and after the participant had rested for at least 5 min. Finally, the average of the last two BP measurements was calculated to determine the BP status of the participant.

Body weight was measured using digital scales (HN-289; Omron, Kyoto, Japan). Subjects were asked to remove shoes and accessories and wear minimal clothing. Height measurement (to the nearest 0.5 cm) was performed without shoes on a standard rigid stadiometer with a mandible plane parallel to the floor. In addition, waist and hip circumferences were also measured. The measurement of waist circumference was taken at the approximate midpoint between the top of the iliac crest and the lower margin of the last palpable rib. On the other hand, hip circumference was measured at the anterior superior iliac spine level or, if this could not be felt, at the widest portion of the buttocks [10]. The measurements were performed using a stretch-resistant tape meter (to the nearest 0.1 cm). The students were required to wear light clothing, and the waist circumference was measured at the end of a normal expiration. All anthropometric measurements were performed by trained nurses.

There were two physicians and two nurses involved in this study, all of whom were trained to conduct data collection procedures following a standardized protocol. The instruments used to measure blood pressure (BP) and anthropometric parameters were calibrated with support from the Vietnam Metrology Institute, located in Hanoi, Vietnam.

### Definitions

Blood pressure classification was based on the JNC 7 guidelines [11]. Hypertension was defined as having systolic BP ≥ 140 mmHg and/or diastolic ≥ 90 mmHg and/or current treatment with antihypertensive medications. Prehypertension was defined as a systolic BP between 120 and 139 mmHg and/or a diastolic BP between 80 and 89 mmHg [11]. Normotension was defined as systolic BP < 120 mmHg and diastolic BP < 80 mmHg. High blood pressure includes those with prehypertension or hypertension.

Body mass index (BMI) was calculated as weight (kg) divided by the square of height in meters (m^2^). According to the WHO diagnostic criteria for overweight and obesity in Asian adults, a BMI between 18.5 and 22.9 kg/m^2^ was classified as normal weight, BMI < 18.5 as underweight, BMI between 23 and 24.9 kg/m^2^ as overweight, and BMI ≥ 25 kg/m^2^ as obese [12]. The waist-hip ratio (WHR) was calculated by dividing the waist measurement (cm) by the hip measurement (cm). Abdominal obesity was defined as a waist-hip ratio ≥ 0.85 for females and ≥ 0.90 for males [10].

Participants were classified as physically inactive if they did not meet the WHO recommendations for physical activity, which included doing at least 150 min of moderate-intensity aerobic physical activity per week or at least 75 min of vigorous-intensity aerobic physical activity per week [13]. Cigarette smokers were defined as participants who reported smoking one or more cigarettes daily for a minimum of six months. Alcohol consumption was defined as participants who reported consuming at least 2 standard drinks per day for males or 1 standard drinks per day for females within the last six months [14]. High salt intake was defined as the habitual addition of salt to meals, as reported by the participant. Low fruit and vegetable consumption was defined as consuming less than three servings of fruit and cooked vegetables (not including pickles) per day. Family history of HTN defined as having a mother, father, sister, or brother who had hypertesion before the age of 60.

### Statistical analysis

Data were entered using the Epidata software version 4.6.0.6, and all statistical analysis was performed by the Stata MP version 17.0 (Stata, College Station, TX, USA). Descriptive statistics were used to analyze the population’s characteristics and the prevalence of PHT and HTN. All continuous variables were presented in the form of mean ± standard deviation (SD) or median (interquartile range) as suitable and compared using the t-test or the Wilcoxon rank-sum test, which depended on the distribution. The categorical variables were presented as numbers (percentages) and compared using the Chi-square (χ2) test or Fisher’s exact test as appropriate. Log-binomial regression was used to explore the significant risk factors of HBP (HTN and PHT) among college freshmen using the prevalence ratio (PR) with a 95% confidence interval (95% CI). The selection of variables in the final model was guided by employing a simplified DAG (**Supplemental Figure** S1). All comparisons were two-tailed, and p-values < 0.05 were considered statistically significant.

## Results

### Description of study population the participants

The baseline characteristics of the male students, female students, and the overall population are presented in Table 1. A total of 840 freshmen (394 men and 446 women) participated in the study; most were 18 years old (90.8%; n = 763). Students with a family history of HTN accounted for 39.7% of the college freshmen, while no one (0%) had a personal history of CVDs before.

**Table 1:** Characteristics of the study participants by sex

Characteristics	Total (n = 840)	Male (n = 394)	Female (n = 446)	*p*-value
Age group (years)				
18	763 (90.8)	353 (89.6)	410 (91.9)	0.242^a^
≥ 19	77 (9.2)	41 (10.4)	36 (8.1)	
**Anthropometric measurements**				
Height (cm)	162.4 ± 8.3	168.6 ± 6.3	156.9 ± 5.3	< 0.0001*^c^
Weight (kg)	52.6 ± 10.2	58.3 ± 10.3	47.6 ± 6.9	< 0.0001*^c^
Body mass index (kg/m^2^)	19.9 ± 2.9	20.5 ± 3.2	19.3 ± 2.5	< 0.0001*^c^
Waist circumference (cm)	67.1 ± 7.7	69.8 ± 8.2	64.7 ± 6.5	< 0.0001*^c^
Hip circumference (cm)	88.8 ± 6.2	90.0 ± 6.8	87.8 ± 5.5	< 0.0001*^c^
Waist-hip ratio	0.75 ± 0.05	0.77 ± 0.05	0.74 ± 0.05	< 0.0001*^c^
**WHO classification**				
Underweight	308 (36.7)	116 (29.4)	192 (43.1)	< 0.001*^a^
Normal	413 (49.2)	190 (48.2)	223 (50.0)	
Overweight	70 (8.3)	50 (12.7)	20 (4.5)	
Obese	49 (5.8)	38 (9.6)	11 (2.5)	
Abdominal obesity	17 (2.0)	5 (1.3)	12 (2.7)	0.144^a^
**Blood pressure**				
Systolic BP (mmHg)	110.8 ± 9.8	115.0 ± 9.3	107.0 ± 8.6	< 0.0001*^c^
Diastolic BP (mmHg)	69.9 ± 7.7	72.9 ± 7.5	67.2 ± 6.9	< 0.0001*^c^
**Blood pressure classification**				
Hypertension	12 (1.4)	10 (2.5)	2 (0.5)	< 0.001*^a^
Prehypertension	281 (33.5)	213 (54.1)	68 (15.2)	
Normotension	547 (65.1)	171 (43.4)	376 (84.3)	
High blood pressure^d^	293 (34.9)	223 (56.6)	70 (15.7)	< 0.001*^a^
**Locality**				
Urban	481 (57.3)	233 (59.1)	248 (55.6)	0.302^a^
Rural	359 (42.7)	161 (40.9)	198 (44.4)	
**Smoking**				
Yes	9 (1.1)	9 (2.3)	0 (0)	0.001*^b^
No	831 (98.9)	385 (97.7)	446 (100)	
**Alcohol consumption**				
Yes	152 (18.1)	116 (29.4)	36 (8.1)	< 0.001*^a^
No	688 (81.9)	278 (70.6)	410 (91.9)	
**Salt intake**				
High	83 (9.9)	39 (9.9)	44 (9.9)	0.987^a^
Adequate	757 (90.1)	355 (90.1)	402 (90.1)	
**Fruit/vegetable consumption**				
Low	668 (79.5)	302 (76.6)	366 (82.1)	0.052^a^
Adequate	172 (20.5)	92 (23.4)	80 (17.9)	
**Family history of hypertension**				
Yes	333 (39.6)	152 (38.6)	181 (40.6)	0.553^a^
No	507 (60.4)	242 (61.4)	265 (59.4)	
**Physical activity**				
Low	461 (54.9)	141 (35.8)	320 (71.7)	< 0.001*^a^
Adequate	379 (45.1)	253 (64.2)	126 (28.3)	

BP: blood pressure, WHO: World Health Organization

^a^: χ2 test; ^b^: Fisher’s exact test; ^c^: *t*-test; ^d^: High blood pressure includes those with prehypertension or hypertension; *: p-value < 0.05

The mean (± SD) BMI of the total was 19.9(± 2.9). The majority (49.2%) of participants were within the normal BMI, while 36.7% were underweight. The prevalence of overweightwas 8.3%, and obesity was 5.8%. Additionally, most participants (98.0%) had a normal waist-hip ratio.

Regarding lifestyle habits, 831 (98.9%) students had no smoking history. Close to one-fifth of the 840 students, 152 (18.1%), reported alcohol consumption. Additionally, 83 (9.9%) students reported that they often added salt to meals. Notably, most participants had a low intake of fruits and vegetables (79.5%), while physical activity was practiced adequately by only 45.1% of the students (Table 1).

### Prevalence of prehypertension and hypertension

The overall prevalence of prehypertension and hypertension was 33.5% (n = 281; 95% CI [30.3–36.8]) and 1.4% (n = 12; 95% CI [0.7–2.5]) among freshmen, respectively, with significant differences between male (54.1%; 2.5%) and female (15.3%; 0.5%) students (p < 0.05; Table 1). Further investigations at VNU Hospital revealed that none of 12 hypertensive students presented with secondary causes such as renal diseases, hypothyroidism, coarctation of the aorta, aldosteronism or pheochromocytoma. As a result, all of them were diagnosed with essential hypertension.

Characteristics of participants according to blood pressure status are presented in Table 2. Participants with high blood pressure had higher BMI (20.8 ± 3.3 vs. 19.4 ± 2.6; p < 0.0001) and waist-hip ratio (0.77 ± 0.05 vs. 0.75 ± 0.05; p < 0.0001). The prevalence rate of alcohol drinking was 13.0% among normal BP students and 27.7% among high BP students (p < 0.001). Moreover, the prevalences of other risk factors (age, smoking, high salt intake, family history of HTN, urban area) were higher in the high BP group, but the differences were non-significant (p > 0.05). Nevertheless, this trend was an exception in the cases of individuals with inadequate physical activity (44.0% in the high BP group vs. 60.7% in the normal BP group).

**Table 2:** Cardiovascular risk factors according to blood pressure status and sex

Risk factors		Total (n = 840)		*p*-value	Male (n = 394)		*p*-value	Female (n = 446)		*p*-value
		Normal BP (n = 547)	High BP (n = 293)		Normal BP (n = 171)	High BP (n = 223)		Normal BP (n = 376)	High BP (n = 70)	
Age > 18 (years)		43 (7.9)	34 (11.6)	0.073^a^	16 (9.4)	25 (11.2)	0.550^a^	27 (7.2)	9 (12.9)	0.109^a^
Body mass index (kg/m^2^)		19.4 ± 2.6	20.8 ± 3.3	< 0.0001*^c^	19.9 ± 3.2	20.9 ± 3.2	0.0019*^c^	19.1 ± 2.1	20.4 ± 3.8	0.0001*^c^
BMI classification	Underweight	231 (42.2)	77 (26.3)	< 0.001*^a^	63 (36.8)	53 (23.8)	0.006*^a^	168 (44.7)	24 (34.3)	0.001*^a^
	Normal	270 (49.4)	143 (48.8)		80 (46.8)	110 (49.3)		190 (50.5)	33 (47.1)	
	Overweight	32 (5.9)	38 (13.0)		19 (11.1)	31 (13.9)		13 (3.5)	7 (10.0)	
	Obese	14 (2.6)	35 (12.0)		9 (5.3)	29 (13.0)		5 (1.3)	6 (8.6)	
Waist-hip ratio		0.75 ± 0.05	0.77 ± 0.05	< 0.0001*^c^	0.77 ± 0.04	0.78 ± 0.05	0.0190*^c^	0.73 ± 0.04	0.75 ± 0.05	0.0198*^c^
Abnormal obesity		11(2.0)	6 (2.1)	0.971^a^	2 (1.2)	3 (1.4)	1.000^b^	9 (2.4)	3 (4.3)	0.413^b^
Urban		316 (57.8)	165 (56.3)	0.684^a^	102 (59.7)	131 (58.7)	0.856^a^	214 (56.9)	34 (48.6)	0.197^a^
Smoking		5 (0.9)	4 (1.4)	0.727^b^	5 (2.9)	4 (1.8)	0.510^b^	0 (0)	0 (0)	-
Alcohol consumption		71 (13.0)	81 (27.7)	< 0.001*^a^	44 (25.7)	72 (32.3)	0.157^a^	27 (7.2)	9 (12.9)	0.109^a^
High salt intake		52 (9.5)	31 (10.6)	0.619^a^	18 (10.5)	21 (9.4)	0.715^a^	34 (9.04)	10 (14.29)	0.177^a^
Low fruit/vegetable consumption		439 (80.3)	229 (78.2)	0.472^a^	132 (77.2)	170 (76.2)	0.823^a^	307 (81.7)	59 (84.3)	0.598^a^
Physical inactivity		332 (60.7)	129 (44.0)	< 0.001*^a^	59 (34.5)	82 (36.8)	0.642^a^	273 (72.6)	47 (67.1)	0.351^a^
Family history of hypertension		207 (37.8)	126 (43.0)	0.145^a^	63 (36.8)	89 (39.9)	0.535^a^	144 (38.3)	37 (52.9)	0.023*^a^

^a^: χ2 test; ^b^: Fisher’s exact test; ^c^: *t*-test; *: p-value < 0.05; BMI: body mass index, BP: blood pressure

### Factors associated with high blood pressure

In Table 3, we present the various risk factors associated with high BP (PHT and HTN) by the log-binomial regression model. Results from the multivariable analyses showed that being male, alcohol consumption and obese were identified to be independent risk factors for high blood pressure (prehypertension and hypertension). Compared to women, men had an approximately three-fold higher prevalence of high blood pressure, which remained statistically significant even after adjustment for age, BMI, waist-hip ratio, locality, family history of HTN, and lifestyle factors (adjusted PR = 3.07, 95% CI [2.32–4.06]). We also observed an association between high BP and BMI categories. Freshmen who were obese had a 35% higher prevalence of high blood pressure compared to those with a normal BMI (adjusted PR = 1.35, 95% CI [1.08–1.68]). In addition, the consumption of alcohol at a minimum of 2 standard drinks per day for males or 1 standard drink per day for females was found to be significantly associated with high BP (adjusted PR = 1.28, 95% CI [1.03–1.59]).

**Table 3:** Risk factors associated with prehypertension/hypertension by log-binomial regression analyses

Risk factors		Bivariable analysis			Multivariable analysis		
		PR	95% CI	*p*-value	aPR	95% CI	*p*-value
Age	per 1 year increase	1.01	0.97–1.24	0.127	1.03	0.92–1.14	0.619
Sex	Male	3.61	2.86–4.55	< 0.001*	3.07	2.32–4.06	< 0.001*
	Female^a^	1.00	–	–	1.00	–	–
BMI classification	Underweight	0.72	0.57–0.91	0.006*	0.80	0.65–1.00	0.052
	Normal^a^	1.00	–	–	1.00	–	–
	Overweight	1.57	1.22–2.02	< 0.001*	1.11	0.87–1.41	0.391
	Obesity	2.06	1.65–2.57	< 0.001*	1.35	1.08–1.68	0.008*
Waist-hip ratio	per 0.05 increase	1.29	1.22–1.36	< 0.001*	1.04	0.95–1.13	0.426
Locality	Urban	0.96	0.80–1.16	0.684	0.85	0.69–1.04	0.113
	Rural^a^	1.00	–	–	1.00	–	–
Smoking	Yes	1.28	0.61–2.67	0.514	0.77	0.38–1.59	0.486
	No^a^	1.00	–	–	1.00	–	–
Alcohol consumption	Yes	1.73	1.44–2.08	< 0.001*	1.28	1.03–1.59	0.026
	No^a^	1.00			1.00		
Salt intake	High	1.08	0.80–1.45	0.613	0.95	0.72–1.24	0.687
	Adequate^a^	1.00	–	–	1.00	–	–
Fruit and vegetable consumption	Low	1.09	0.87–1.35	0.467	1.04	0.87–1.25	0.660
	Adequate^a^	1.00	–	–	1.00	–	–
Physical activity	Low	0.65	0.54–0.78	< 0.001*	1.07	0.90–1.26	0.438
	Adequate^a^	1.00	–	–	1.00	–	–
Family history of hypertension	Yes	1.15	0.95–1.38	0.143	1.09	0.93–1.28	0.279
	No^a^	1.00	–	–	1.00	–	–

^a^: Reference group; *: p-value < 0.05; aPR: adjusted prevalence ratio; BMI: Body mass index, CI: Confidence interval

## Discussion

High blood pressure is a public health challenge worldwide, and it has emerged as a significant medical problem among adolescents and young adults [5, 7, 15]. In numerous developed countries, the prevalence of hypertension and prehypertension has been investigated across various age groups. In contrast, similar data are lacking in Vietnam, particularly for youth around the age of 18 years. To the best of our knowledge, this is the first study to report the prevalence of prehypertension/hypertension and related risk factors among college freshman students in Vietnam.

In this cross-sectional study, the prevalence of prehypertension was 33.5% among VNU freshmen, which was similar to the rate in the university student population of Thailand (32.8%), Bangladesh (37.8%), Ethiopia (35.7%), Kuwait (39.5%) and higher than the rate in Indonesia (11.3), Laos (19.4), Malaysia (11.5), Philippines (14.5), Myanmar (18.6%), Uganda (18.8%), and Palestine students (27.1%) [9, 16–20]. In addition, our study’s overall prevalence rate of hypertension was 1.4%, comparable to that in Palestine (2.2%), Philippines (3.2%), Malaysia (3.2%), Laos (3.9%), and China (4.3%), but significantly lower than that reported in Indonesia (11.3), Thailand (32.8%), Bangladesh (10.2%), Ethiopia (7.7%), Kuwait (7.0%) and other previous studies [9, 16–22]. Notably, among the 293 students with PHT/HTN in our study, 275 (94%) of them had not previously undergone blood pressure screening. This can be attributed to the scarcity of preventive healthcare resources, a prevailing issue in Vietnam, where students typically do not undergo comprehensive health examinations until they have noticeable symptoms. Such circumstances require the appropriate attention of all stakeholders in education, which includes the government, parents, students, school authorities, and lecturers, to ensure the effectiveness of preventive health programs.

In the last three decades, a growing body of epidemiological research has revealed a positive correlation between being overweight and having high blood pressure in young populations, which indicated that obesity was an important risk factor for elevated blood pressure [23–25]. The enhancement of living conditions in Vietnam has resulted in a rise of obesity, particularly among young individuals [26, 27]. Our data showed that the prevalence of overweight/obesity was as high as 14.1% (8.3% and 5.8%) in young VNU students, which was in line with previous reports in other developing countries [20, 28]. The positive and significant association between overweight/obesity and high blood pressure has been shown in several studies [16, 21, 29–31]. The present study found that prehypertension and hypertension were more common among people who were overweight or obese in both men and women. Moreover, multivariable log-binomial regression analysis indicated that obese students had roughly threefold increased prevalence of having high blood pressure compared to those with a normal BMI, which aligns with previous findings [18, 19]. These findings are consistent with the results from earlier studies [32–34]. On the other hand, the pathophysiology of hypertension in patients with obesity is complex and still an area of research. Nevertheless, mechanisms involved in the pathogenesis of obesity-induced hypertension may include insulin resistance, hyperleptinemia, endothelial and vascular dysfunction, neuroendocrine imbalances, sympathetic nervous system hyperactivity, structural and functional renal changes, maladaptive immune and inflammatory responses, and stimulation of the renin-angiotensin-aldosterone system [35–38]. Additionally, hypertension development in the presence of obesity may also be attributed to alterations in adipose-derived cytokines and uric acid [35]. Our results once again indicate that modifying lifestyle, such as decreasing fat content in the diet and increasing physical activity to achieve weight loss, may be an efficient approach to lower blood pressure in the long term for young adults [39].

In our study, men had a more than 3-fold higher prevalence of high blood pressure (prehypertension and hypertension) than women (56.6% vs. 15.7%). Moreover, the multivariable log-binomial regression analyses showed that the male sex was an independent risk factor for high BP. Recently, a prior study conducted in Bangladesh also revealed a higher prevalence of prehypertension/hypertension among male students (60.0% vs. 25.3%) [19]. A similar result for high blood pressure prevalence among university students (41.3% vs. 11.6%) was reported by another survey carried out in Palestine [9]. The findings of this study also align with the results of a study on young people in Kuwait, where it was discovered that hypertension is more prevalent among male students (72.8%) than female students (26.5%) [16]. Although the exact mechanism behind this sex disparity has not yet been determined, some reliable evidence suggests that androgens may be involved in regulating blood pressure differences between the sexes [40]. In addition, estrogen production during the premenopausal period in women induces vasodilation by promoting the release of endothelium-derived relaxing substances, such as nitric oxide and prostacyclin, or through its direct effect on the vascular smooth muscle. This may serve as protection against the development of hypertension [41].

Alcohol consumption is prevalent among a global population exceeding 2 billion individuals [42]. This widely consumed substance is frequently subjected to abuse, and its usage has been associated with a broad range of adverse health effects, encompassing more than 200 disorders, including hypertension [42]. In the present study, alcohol consumption was found to be significantly associated with high BP (adjusted PR = 1.28, 95% CI [1.03–1.59]). This finding was similar to some previous studies in young adults [43–45]. Alcohol’s impact on blood pressure can be attributed to various mechanisms. These mechanisms encompass cellular impairments leading to arterial plaque accumulation, disturbances in arterial-vascular function, as well as hormonal imbalances affecting the body’s fluid and blood pressure regulation through the renin-angiotensin-aldosterone system (RAAS) [46]. In addition, alcohol can also trigger several other adverse mechanisms, including alterations in intracellular calcium levels, changes in heart rate, disruptions in baroreflex control, endothelial dysfunction, vascular wall oxidative stress, and activation of other neurohormonal systems, such as the sympathetic nervous system, in addition to the RAAS [47].

Unlike several previous studies, the associations between high BP and insufficient physical activity, high sodium intake, and low fruit/vegetable consumption were not found in our study [22, 30, 48, 49]. However, several other studies also did not detect a significant association between them and elevated blood pressure among young students [9, 17–19, 50]. It’s possible that the cause of the rise in blood pressure in our study group is not only due to lack of physical activity and low intake of fruits and vegetables, but it could also be related to other factors such as being overweight [18, 50]. Moreover, some research has shown that there is a strong correlation between high BP and sodium intake in older individuals but not in young people, then factors other than sodium intake, such as being overweight, may have a more significant impact on elevated blood pressure in young adults [51–53]. On the other hand, the possibility of exposure misclassification arises when utilizing self-reported surveys for data collection. Consequently, the association between lifestyle risk factors and high BP may lean towards null, necessitating cautious interpretation of the findings.

Interestingly, smoking was not significantly associated with high blood pressure in this study after adjusting for confounders. This finding was not comparable to some previous studies [22, 54]. However, some other studies showed similar results to our study [17, 19, 20, 55]. The difference was probably related to several reasons. First, the differences in population characteristics, such as our study’s inclusion of college freshmen with a younger age, and the variations in smoking definitions, may contribute to the reported disparities in associations across different studies. Second, underreporting of smoking may have occurred because of social disapproval, being stigmatized or self-reporting bias. Third, it is recognized that this factor may take a longer period of time to have an effect on blood pressure [56]. Finally, participants in our study were college freshers, which have.

Despite the seemingly low prevalence of hypertension, the high prevalence of prehypertension indicates that paying more attention to early preventive behavioral strategies targeted explicitly toward Vietnamese youth is necessary. Futhermore, the implementation of comprehensive regular health check-ups and the promotion of healthy (such as a healthy diet, adequate physical activity, smoking cessation, and limiting alcohol intake) as well as fostering self-awareness regarding health protection among students can also make a significant contribution to the early detection and prevention of cardiovascular disorders, particularly PHT/HTN.

### Study strengths and limitations

The major strength of this study was that it gathered data on both sexes from Vietnam’s largest public university. Another strength of this study is that most of the demographic and anthropometric variables that could be associated with elevated blood pressure were investigated. Furthermore, most of the participants were 18 years old, which made them more vulnerable to risk factors, but they had insufficient awareness about disease prevention. This study contributed to the evidence on preventive cardiology and emphasized the importance of early prevention of hypertension.

However, the present study also had some limitations. First, this was a cross-sectional study and failed to establish the causal relationship between risk factors and the development of PHT and HTN. Second, all the information collected in the study, except anthropometric and blood pressure measurements, was based on self-reporting. Certain behaviors could have been underreported due to the recall bias. Third, due to limitations in economic and human resources, the data including homocysteine levels, blood lipid, and blood glucose, which may also influence hypertension development, were lacking. Finally, it is important to note that our research was conducted within a university setting, where the socioeconomic status (SES) of the study population may significantly differ from that of young individuals outside the university, who may have lower education levels and/or come from families with lower economic statuses. As a result, the generalizability of the findings may be limited by this variation in SES. Therefore, futher studies are necessary to validate the results among the broader non-university young population.

## Conclusions

The results of this study revealed the high prevalence of prehypertension/hypertension among young students in Vietnam. A high rate of overweight and physical inactivity was also indicated. Male sex, alcohol consumption, and obesity were identified as significant risk factors for prehypertension/hypertension among young students. The results of our study highlight the importance of promoting a healthy lifestyle and screening for obesity and high blood pressure among young freshmen in order to control modifiable CVD risk factors. A large longitudinal study might be more effective in assessing the prevalence of prehypertension/hypertension among young adults and all their related factors nationally.

## Electronic supplementary material

Below is the link to the electronic supplementary material.

Supplementary Material 1

Supplementary Material 2

## Data Availability

The data that support the findings of this study are not openly available due to reasons of sensitivity and are available from the corresponding author upon reasonable request.

## Abbreviations

BMI: Body mass index
BP: Blood pressure
CI: Confidence interval
CVD: Cardiovascular diseases
HBP: High blood pressure
HTN: hypertension
PR: Prevalence ratio
PHT: Prehypertension
VNU: Vietnam National University, Hanoi
WHO: World Health Organization
