# Supplementary figures and images for: Prevalence and risk factors of prehypertension/hypertension among freshman students from the Vietnam National University: a cross-sectional study
Source: BMC Public Health. 2023 Jun 16;23:1166. doi: 10.1186/s12889-023-16118-4 (PMC10276403; doi:10.1186/s12889-023-16118-4)

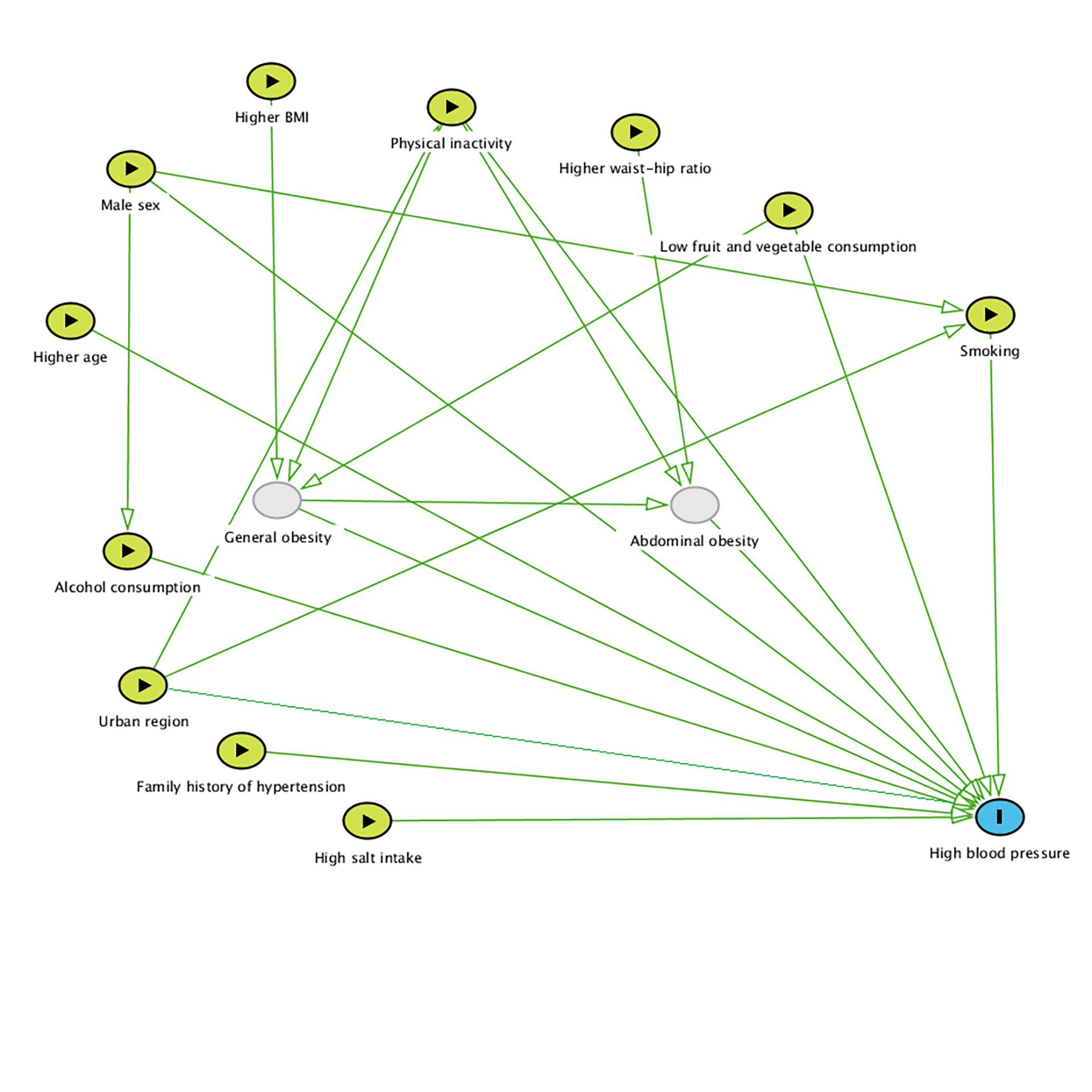

Supplement: Supplementary file 1 — Supplementary Material 1 [file 12889_2023_16118_MOESM1_ESM.png]
